# Higher IL-6 and IL6:IGF Ratio in Patients with Barth Syndrome

**DOI:** 10.1186/1476-9255-9-25

**Published:** 2012-06-21

**Authors:** Lori D Wilson, Sadeeka Al-Majid, Cyril S Rakovski, Christina D Schwindt MD

**Affiliations:** 1 Department of Pediatrics Center, University of California, Irvine, Irvine, 101 The City Drive, Bldg 25, 2nd Floor, Orange, CA, 92868, USA; 2 California State University, Fullerton, School of Nursing, 6868, Fullerton, CA, 92834-6868, USA; 3 Chapman University, Schmid College of Science and Technology, Chapman University, 545 West Palm, Orange, CA, 92866, USA; 4 Department of Pediatrics, University of California, Irvine, 101 The City Drive, Bldg, 55, 3rd Floor, Orange, CA, 92868, USA; 5 Department of Kinesiology, California State University, Bellflower Boulevard, Long Beach, CA, 90840, USA; 6 Southern California Research, 27800 Medical Center Road, Mission Viejo, CA, 92691, USA

**Keywords:** Myopathy, dilated-cardiac myopathy, inflammation, catabolic, cardiolipin, mitochondria

## Abstract

**Background:**

Barth Syndrome (BTHS) is a serious X-linked genetic disorder associated with mutations in the tafazzin gene (TAZ, also called G4.5). The multi-system disorder is primarily characterized by the following pathologies: cardiac and skeletal myopathies, neutropenia, growth delay, and exercise intolerance. Although growth anomalies have been widely reported in BTHS, there is a paucity of research on the role of inflammation and the potential link to alterations in growth factors levels in BTHS patients.

**Methods:**

Plasma from 36 subjects, 22 patients with Barth Syndrome (0.5 - 24 yrs) and 14 healthy control males (8 - 21 yrs) was analyzed for two growth factors: IGF-1 (bound and free) and Growth Hormone (GH); and two inflammatory cytokines IL-6 and TNF-α using high-sensitivity enzyme-linked immunosorbent assays.

**Results:**

The average IL-6 and IL6:IGF ratio levels were significantly higher in the BTHS (p = 0.046 and 0.02 respectively). As for GH, there was a significant group by age interaction (p = 0.01), such that GH was lower for BTHS patients under the age of 14.4 years and higher than controls after age 14.4 years. TNF-α levels were not significantly different, however, the TNF-α:GH was lower in BTHS patients than controls (p = 0.01).

**Conclusions:**

Comparison of two anabolic growth mediators, IGF and GH, and two catabolic cytokines, IL-6 and TNF-α, in BTHS patients and healthy age-matched controls demonstrated a potential imbalance in inflammatory cytokines and anabolic growth factors. Higher rates of IL-6 (all ages) and lower GH levels were observed in BTHS patients (under age 14.5) compared to controls. These findings may implicate inflammatory processes in the catabolic nature of Barth Syndrome pathology as well as provide a link to mitochondrial function. Furthermore, interactions between growth factors, testosterone and inflammatory mediators may explain some of the variability in cardiac and skeletal myopathies seen in Barth Syndrome.

## Background

Barth Syndrome (BTHS) is a serious X-linked genetic disorder associated with mutations in the tafazzin gene (TAZ, also called G4.5). This multi-system disorder is primarily characterized by the following pathologies: cardiomyopathy (dilated or hypertrophic), neutropenia (chronic, cyclic, or intermittent), hypotonia and muscle weakness, growth delay, exercise intolerance, cardiolipin abnormalities, and 3-methylglutaconic aciduria. Barth Syndrome is believed to be severely under-diagnosed and is estimated to occur in one out of approximately 300,000 births[1]. Being able to analyze plasma from 22 BTHS patients against health control subjects in such a rare disease population is noteworthy strength of this study.

Although growth anomalies have been widely reported in BTHS, there is a paucity of research on the contribution of catabolic/anabolic processes, the influence of inflammation and the potential link to alterations in growth factor levels in BTHS patients. Recently, however there has been growing evidence that inflammatory processes may influence normal muscle development in children. Increased levels of Tumor Necrosis Factor alpha (TNF-α) have been shown to suppress the AKT/mTOR (mammalian target of rapamyosin) pathway, a crucial pathway for regulating skeletal muscle hypertrophy and thereby increase muscle catabolism [2-4]. Inflammatory cytokines may also antagonize the anabolic effects of Insulin-like growth factor (IGF), a known promoter of muscle hypertrophy [5-7]. Normal levels of physical activity have been linked to a balance between anabolic factors such as IGF-1 and Growth Hormone (GH) and catabolic cytokines such as Interleukin-6 (IL-6) and TNF-α. For example, higher levels of IL-6 and lower levels of IGF-1 have also been observed in children with chronic inflammatory diseases such as juvenile idiopathic arthritis, inflammatory bowel disease and cystic fibrosis [8,9]. Thus it is plausible that a catabolic/anabolic imbalance, linked to an inflammatory process, contributes to the growth abnormalities and pathology observed in BTHS.

We hypothesized that patients diagnosed with BTHS would have an imbalance in catabolic and anabolic mediators such that BTHS would have lower levels of growth factors and higher levels of inflammatory mediators compared to age-matched healthy controls. This study addresses this question by statistical analysis of IGF-1, GH, IL-6 and TNF-α plasma levels obtained from BTHS patients and healthy controls.

## Methods

### Sample characteristics

The sample population for this study included 36 subjects, 22 BTHS patients (age 4 months to 24 yrs) and 14 healthy controls (age 8 to 21 years). Plasma and clinical information from the BTHS patients was provided by the Barth Syndrome Foundation Bioregistry. Plasma from healthy controls was obtained from subjects participating in studies conducted through the Pediatric Department at the University of California, Irvine (UCI). All subjects were knowing and willing plasma donors as members of Barth Syndrome Foundation or from IRB-approved studies at UCI. The UCI studies involved children age eight years and older, therefore plasma samples were not available from for healthy controls below age eight. Exclusion criteria for healthy controls included having had an upper respiratory infection or inflammatory illness such as asthma. Furthermore, healthy controls subjects had not utilized any antibiotics or non-steroidal anti-inflammatory (NSAID) medications prior to the study (14-days for antibiotics and 7-days for NSAIDs).

### Blood acquisition, processing and cytokine measurement

Plasma from both the BTHS patients and healthy controls was obtained via standard phlebotomy procedures. Within two hours of acquisition, blood was centrifuged and stored at −80 °C. Barth Syndrome samples were shipped on dry ice and immediately stored at −80 °C so that they would be thawed only once for analysis.

Cytokines and growth mediators were measured using high-sensitivity Immunoassay quantification kits, as follows: IL-6 and TNF-α were measured using kits by R&D Technologies, IGF-1 (bound and free) was measured using a DSL kit, and human Growth Hormone was measured using the RayBiomed kit. The sensitivity of the tests were: IL-6 (0.016 pg/mL), TNF-α (0.038 pg/mL), IGF-1 (0.015 ng/mL) and GH (4.0 pg/mL). All samples were run on a single 96- well plate. Duplicates were run for randomly selected BTHS patients and healthy controls to fill all wells of the single plate.

### Statistical analysis

We implemented linear regression modeling to find the sets of significant predictors for all outcomes variables of interest and quantify the corresponding effect sizes. All study variables were considered in the model building process as potential independent predictors or confounders with main covariates of interest being age, case–control status (BTHS versus Healthy Controls), and their interaction. Thus, separate best predictive models were derived and assessed for goodness of fit for IL-6, IGF1-β, GH, absolute neutrophil count, as well as the ratios between IL-6 and IGF-1 (IL6:IGF), and TNF and GH (TNF:GH). For models in which age revealed a nonlinear effect on the outcome variable, a discretized version of age (puberty) was used with interval allocation, 0–7, 8–11, 12–16, and 18–21 years of age.

All computational steps were carried out using the R statistical software language (R Development Core Team (2008) [10].

## Results

### Demographic characteristics

Demographic data are presented in Table 1. As stated previously, matched controls were not available under the age of eight years. Height and weight data were missing for the following five BTHS patients: two age 8–11 years, one age 12–16 years, and two age 18 to 24 years. Age and height did not differ significantly between controls and BTHS patients within the age groups; however, the BTHS patients had significantly lower body weight than healthy controls (p < 0.001).

### Catabolic and anabolic mediator levels

**Figure 1:**
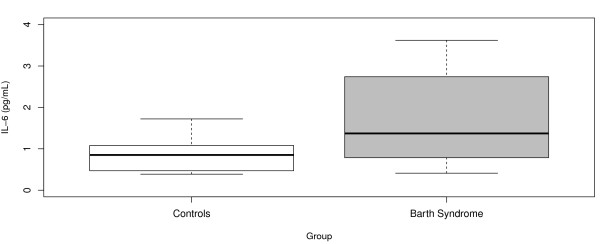
**IL-6 in Barth Syndrome Patients and Healthy Controls.** Box Plot distribution of circulating IL-6 levels in Controls and BTHS patients. IL-6 levels were significantly higher across all age groups, (p = 0.046).

There was no effect of age on IL-6 (both linear and nonlinear trends were considered and tested) and the only significant predictor was BTHS status (details given in Table 2). Our results show that on average, the BTHS group possessed significantly higher IL-6 levels (p = 0.046) throughout their lives compared to the healthy controls with mean IL-6 values of 3.19 and 0.81 pg/mL respectively. Graphical comparison of the two distributions is presented in Figure 1. The *R*^2^ value for this model was 0.13 reflecting the inherent variability in the IL-6 measurements.

**Figure 2:**
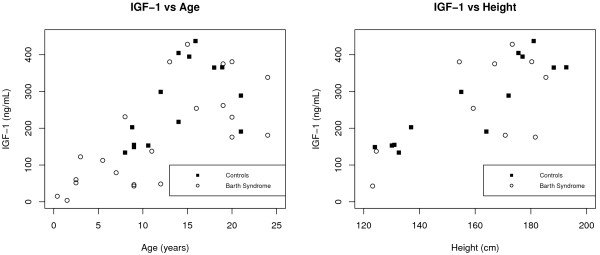
**Predictors of IGF-1.** Both Age (Figure to Left) and Height (Figure to Right) were correlated with IGF-1 level. Changes across Age and Height did not differ between BTHS (open circles) and Controls (closed boxes).

With respect to IGF-1, the mean IGF-1 levels for the Controls and BTHS Patients were 286.39 and 197.90 ng/mL respectively (p = 0.05). This difference was found to be spurious, however, with linear regression since it was driven by the underlying contribution of differences in the distributions of age and height between the two groups. This is evidenced by the fact that after adjustment for height and age, the case–control status becomes highly non-significant (p = 0.46). We found that height, age, and the height * age interaction were significant predictors of IGF-1 levels. After controlling for these factors, having BTHS no longer played a significant role in the prediction of IGF-1 (details given in Table 3). The presence of the interaction term between two continuous variables makes the relationship between IGF-1 and height and age difficult to interpret. However, the negative sign of interaction accounts for the decrease of the IGF-1 levels after age 17 (scatterplots of both the height and age variables versus IGF-1 are presented in Figure 2. The *R*^2^ value for this model was 0.79 indicating a high level goodness-of-fit.

**Table 1:** Demographic Data on Study Sample Population

	Age Group (years)							
	Healthy Controls by Age Group (mean ± SD (n))				BTHS by Age Group (mean ± SD (n))			
Variable	0.5 to 7	8 to 11	12 to 16	18 to 21	0.5 to 7	8 to 11	12 to 16	18 to 24
Age (yrs)	~	9.1 ± 0.9(5)	14.2 ± 1.5(5)	19.7 ± 1.5(4)	3.2 ± 2.3(7)	9.3 ± 1.3(4)	14.0 ± 1.8(4)	20.9 ± 2.2(7)
Height (cm)	~	130.9 ± 4.7(5)	172.1 ± 11.7(5)	179.2 ± 13.5(4)	~	23.9 ± 0.9(2)	162.3 ± 9.8(3)	177.0 ± 7.8(5)
Weight (kg)	~	28.1 ± 3.1(5)	69.7 ± 19.7(5)	75.2 ± 5.1(4)	~	21.9 ± 1.1(2)*	45.6 ± 3.1(3)*	56.6 ± 15.4(5)*

* Indicates statistically significant difference in weight (p < 0.001).

~ Indicates missing data.

**Table 2:** Model Summary of the Significant Predictors of IL-6

Variable	Estimate	Standard Error	t-value	p-value
Intercept	0.81	0.93	0.87	0.39
Group (BTHS vs. Control)	2.38	1.18	2.01	0.046

**Table 3:** Model Summary of the Significant Predictors of IGF-1

Variable	Estimate	Standard Error	t-value	p-value
Intercept	−1379.60	382.32	−3.61	0.002
Height (cm)	11.71	2.48	4.71	<0.001
Age (years)	56.02	31.37	1.79	0.09
Height * Age	−0.42	0.19	−2.27	0.03

**Figure 3:**
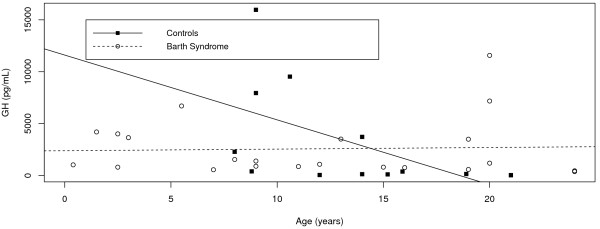
**Changes in GH by Group over Time.** Scatter plot of circulating GH level by Age for BTHS (open circles) and Controls (closed boxes). Interaction can be seen at the point in which the slope of the BTHS patients (dashed line) crosses the Controls (solid line). BTHS patients had significantly lower GH levels before the age 14.5 years.

With respect to GH, we found that the significant predictors were BTHS status, age and their interaction (details given in Table 4). The presence of the interaction term between the continuous variable age and factor variable denoting Barth Syndrome status defines a model that incorporates different slopes and different intercepts for the BTHS and Control group (scatterplot and regression lines are presented in Figure 3). The BTHS patients have lower GH levels compared to healthy controls for ages younger than 14.4 years and higher GH levels for ages older than 14.4 years. As GH is known to be secreted in several pulses or peaks during the day, with wide variations between days and individuals, these naturally occurring fluctuations can introduce variability, which was reflected in the *R*^2^ value of 0.19 for this model.

**Table 4:** Model Summary of the Significant Predictors of GH

Variable	Estimate	Standard Error	t-value	p-value
Intercept	11602.30	3313.10	3.50	0.001
Group (BTHS vs. Control)	−9216.00	3598.20	−2.56	0.02
Age (years)	−624.30	236.20	−2.64	0.01
Group * Age	639.90	256.60	2.49	0.01

With respect to IL-6:IGF-1 ratio, we found that there was no effect of age (both linear and nonlinear trends were considered and tested) and the only significant predictor was BTHS status (details given in Table 5). Our results show that on average, Barth Syndrome patients possessed significantly higher IL-6:GF-1 ratios (p = 0.03) throughout the age groups analyzed compared to the healthy controls with mean.

**Table 5:** Model Summary of the Significant Predictors of IL-6:IGF-1

Variable	Estimate	Standard Error	t-value	p-value
Intercept	0.003	0.011	0.31	0.76
Group (BTHS vs. Control)	0.031	0.014	2.22	0.03

**Figure 4:**
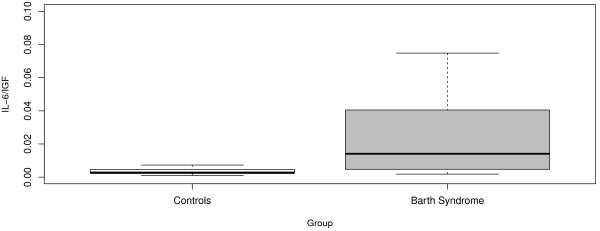
**IL-6:GF-1 Ratio in BTHS and Controls.** Box plot distribution of IL-6:IGF-1 ratio BTHS patients and Controls. The IL-6:IGF-1 ratio was significantly higher in BTHS patents across all age groups (p = 0.03).

IL-6:IGF-1 ratios of 0.003 and 0.034 respectively. Graphical comparison of the two distributions is presented in Figure 4. The *R*^2^ value for this model was 0.13 reflecting the inherent variability in the IGF-1 measurements induced by IL-6.

**Figure 5:**
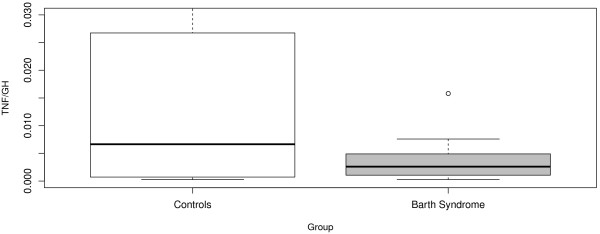
**TNF:GH Ratio in BTHS and Controls.** Box plot distribution of TNF:GH ratio for BTHS patients and Controls. The TNF:GH ratio was significantly higher in Controls across all age groups (p = 0.02).

Lastly, with respect to TNF:GH ratio, we found that there was no effect of age (both linear and nonlinear trends were considered and tested) and the only significant predictor was Barth Syndrome status (details given in Table 6). Our results show that on average, the Barth Syndrome group possessed significantly lower (p = 0.02) TNF:GH ratios throughout their lives compared to the healthy controls with mean TNF:GH ratios of 0.004 and 0.019 respectively. Graphical comparison of the two distributions is presented in Figure 5. The *R*^2^ value for this model was 0.17 reflecting the inherent variability of the TNF:GH ratio induced by both TNF and GH values.

**Table 6:** Model Summary of the Significant Predictors of TNF:GH

Variable	Estimate	Standard Error	t-value	p-value
Intercept	0.019	0.004	3.93	<0.001
Group (BTHS vs. Control)	−0.015	0.006	−2.52	0.02

## Discussion

Lower levels of two anabolic growth mediators, (IGF and GH), and a higher level of the catabolic cytokine IL-6 were observed in this study and point to an imbalance which could contribute to some of the myopathies and growth delays present in BTHS patients. Higher levels of IL-6 have been found in sedentary children and adolescents as well as in chronic diseases of childhood [11,12]. Indeed, the levels of IL-6 observed in this group of BTHS patients were similar to those reported in children with Type I diabetes mellitus [13], suggesting the contribution of a low-grade inflammatory process. Thus two potential explanations for the higher IL-6 levels in Barth Syndrome include the lower levels of physical activity resulting from exercise intolerance and low grade inflammatory processes in Barth Syndrome. This study demonstrates, for the first time, a catabolic/anabolic imbalance, with a link to chronic inflammation, in BTHS patients which may explain the growth abnormalities and myopathies associated with this syndrome.

The present case is a complex and sophisticated example of the importance of implementing regression modeling in the statistical analysis of IGF-1. While it appeared as if IGF-1 was lower in the BTHS patients (mean IGF-1 for Controls = 286.4 ng/mL and BTHS 197.90 ng/mL respectively, p = 0.05). This difference was found to be spurious with linear regression since it was driven by the underlying contribution of differences in the distributions of age and height between the two groups. After adjusting for height and age, the case–control status was found not be significant (p = 0.46). This is a clear example in which regression modeling detects the true predictors of IGF-1 levels while direct approaches such as correlation analysis would have been inaccurate. The authors suggest that further investigation of IGF-1 be conducted and that study subjects be age and height matched so that the exact contribution of case control status can be identified.

Growth delays are widely recognized in BTHS with accelerated to normal growth occurring during the mid- to late- teenage years. IGF is well known to be closely correlated with growth in adolescents [14]. Moreover, numerous studies demonstrate that IGF levels predict both height and weight [15,16]. We also found this to be true for both the BTHS patients as well as the healthy controls. In the BTHS patients, IGF was highly correlated with both height and weight (bivariate correlations, *R*^2^ = 0.77 for height and *R*^2^ = 0.71 for weight). Thus, IGF signaling for bone growth appeared to be “normal” in the BTHS patients. This was despite significant differences in weight; (lower weights in BTHS). We hypothesize that the lower weights were due to reduce muscle development and not bone growth in the BTHS patients. A possible explanation for the reduced muscle mass in BTHS patients is differences in the mediator milieu of the muscle during early development. This is supported by studies demonstrating that cell signaling in the muscle is influenced by the mediator environment. Adams and colleagues showed differences in cell signaling in the muscle of rats based upon either acute or chronic exposure to IL-6 [17]. Chronic IL-6 exposure reduced muscle growth whereas acute exposure occurring with regular exercise training enhanced muscle growth. Indeed, differences in mediators during early development may also account for the delay in bone growth observed in BTHS patients.

The achievement of normal height in adolescent BTHS patients is an intriguing occurrence and a phenomenon that is not unknown in pediatrics. It is well recognized that children suffering from malnutrition or with health problems causing poor growth have “catch-up growth.” Catch-up growth is a growth spurt that occurs when normal conditions are restored. Recently, IGF has been shown to be involved in catch-up growth [18]. In a study of zebrafish, in which growth delays occurred from oxygen deprivation but catch-up growth occurred following restoration of oxygen, it was demonstrated that catch-up growth could be blocked by blocking IGF. The specific pathway involved is the IGF-MAP kinase pathway; however, it may not be the only pathway that figures in, and the specific pathway used may depend on circumstances.

Additionally, other mechanisms may be are implicated in catch-up growth in BTHS patients. For example, we found that GH levels increased after the age of 14.4 years in BTHS and another potential explanation is the coincidental pubertal-related increase in testosterone. Testosterone is known to increase the effectiveness of IGF signaling via improvement in binding protein interactions (i.e. IGF BP 4 and 5)[14]. This has the potential to synergistically impact GH expression and activation because cells which were previously resistant to IGF or GH may become more sensitive to increases via binding proteins. This may be from testosterone, IL-6 or from completely unidentified mechanisms. For example, infusion of IL-6 has also been shown to increase IGF sensitivity in acute scenarios [17]. Most recently, convergent mechanism for both cytokines and growth factors in cell signaling in skeletal muscle have been elucidated [18]. Transcription factors have been identified that demonstrate overlap between IL-6 and IGF in the cell replication cycle include: Janus kinase/signal transducer activator of transcription (JAK/STAT) pathway and suppressors of cytokine signaling (SOCS). Weigert et al. found that acute treatment with IL-6 failed to stimulate increases in SOC3 expression; however, Adams et al. found that long-term exposure elevated levels of SOCS3 mRNA[17,19]. Thus, several factors may be involved in the growth delay seen in BTHS and subsequent catch-up growth.

The influence of IL-6 on growth may involve a paradoxical effect. Indeed, the role of IL-6 in muscle has been shown to demonstrate an intriguing paradox [20]. Exercising muscle is known to increase IL-6 production; while at the same time elevated IL-6 expression is often found during muscle wasting conditions [21]. In the BTHS patients, circulating IL-6 levels were significantly higher across all age groups (neither age nor pubertal status were significant). Since BTHS patients are exercise intolerant, there are two possibilities: 1) that normal daily activities in the BTHS patients mimic intense exercise in the muscles of healthy controls, i.e. normal activity increases circulating IL-6 in Barth Syndrome, or 2) that IL-6 is being secreted at higher levels by immune cells (possibly dysfunctional neutrophils) in Barth Syndrome patients relative to healthy controls. These hypotheses offer two valuable areas of research in the area of IL-6 production by muscle and/or immune cell.

Most recently, convergent mechanism for both cytokines and growth factors in cell signaling in skeletal muscle have been elucidated [21]. Transcription factors have been identified that demonstrate overlap between IL-6 and IGF in the cell replication cycle include: Janus kinase/signal transducer activator of transcription (JAK/STAT) pathway and suppressors of cytokine signaling (SOCS). Weigert et al. found that acute treatment with IL-6 failed to stimulate increases in SOC3 expression; however, Adams et al. found that long-term exposure elevated levels of SOCS3 mRNA.

Duan and colleagues also demonstrated that altering IGF in muscles alters growth [22]. In this study, age was significantly correlated with IGF-1 but not GH (r =−0.16, p = 0.36). It is possible that the delayed peak in GH is contributory to the growth delays observed in Barth Syndrome. Moreover, there is growing evidence linking GH levels with mitochondrial damage [23,24]. It has been proposed that lower levels of circulating GH may limit mitochondrial uptake of GH and contribute to the mitochondrial damage such as is associated with Barth Syndrome. It has been shown that extracellular supplies of GH are taken up and localize to the mitochondria [23,24]. These findings provide a potential link between reduced growth mediator levels and mitochondrial dysfunction.

The IGF "axis" is also commonly referred to as the Growth Hormone/IGF1 Axis. IGF-1 is mainly secreted by the liver as a result of stimulation by GH. The diurnal variation of GH is well documented. For this reason, IGF and its binding proteins are often measured simultaneously to assess growth delays and gigantisms because they are stable over time [25].

One of the most interesting findings of this study involved the link between IL-6 and IGF-1, specifically the significantly higher IL-6:IGF-1 ratio in BTHS patients. There is increasing evidence that inflammation contributes to the necrosis of dystrophic myofibers in Duchenne muscular dystrophy[7]. IGF plays a central role in myofibril hypertrophy and atrophy and this balance is of critical importance in sarcopenia, cachexia, and metabolic syndrome [26,27]. The combination of both a slight increase in IL-6 and small decrease in IGF-1 may have a more substantial contribution to muscle and cardiac myopathies than previously considered in BTHS.

Contrary to the higher IL-6:IGF ratio in BTHS, the TNF-α:GH ratio was significantly lower in Barth patients than controls in the older age groups. A study controlling for diurnal variation in GH would be important to understand the clinical implications of this finding, in particular because there were not differences in TNF-α by comparison group. In regards to the TNF-α:IGF ratio, age was a highly significant predictor of the differences in this catabolic:anabolic ratio outcome (p = 0.004) in this study. After controlling for age, however, the group effect (BTHS vs. control) was not significant (p = 0.236). The relationship to these two factors remains of interest since it has been shown that IGF-1 was preventative of and TNF-α enhanced myocardial injury after ischemia/reperfusion following myocardial infarction [28].

A limitation of this study includes the lack of full control and potential variation in the time of day plasma collection from BTHS patients. Also the amount of time between acquisition and storage may have differed within the sample. Sack and colleagues found that plasma concentrations of TNF-α were remarkably unstable and that it should be measured immediately post blood draw [29]. Sacks also found that TNF levels were highly variable during childhood and should be analyzed with great precaution.

Another weakness is the small sample size and cross-sectional nature of the study. We believe, further investigation into the role of TNF-α in Barth Syndrome cardiac and skeletal myopathies is warranted in a larger and more well-characterized group.

## Conclusion

Comparison of two anabolic growth mediators, IGF and GH, and two catabolic cytokines, IL-6 and TNF-α, in Barth Syndrome patients and healthy age-matched controls demonstrated a possible catabolic:anabolic imbalance in Barth Syndrome. Higher rates of IL-6 and lower IGF-1 levels were observed in BTHS compared to controls. This finding may implicate inflammatory processes in the catabolic nature of Barth Syndrome pathology as well as provide a link to mitochondrial dysfunction. Furthermore, lower levels of IGF-1 may contribute to some of the growth delays and myopathies observed in Barth Syndrome.

## Competing interest

The authors declare that they have no competing interests.

## Authors’ contributions

LDW and CS developed the research hypothesis and study methodologies, LDW carried out the laboratory analyses, CR conducted the statistical analyses, and SA assisted in the interpretation of findings. All authors contributed toward the writing of the final manuscript.
